# 4-(3-Fluoro­phen­yl)-6-hy­droxy-5-(thio­phen-2-ylcarbon­yl)-6-trifluoro­methyl-1,3-diazinan-2-one

**DOI:** 10.1107/S1600536811006933

**Published:** 2011-03-02

**Authors:** Qin He, Jing Li, Bao-Jun Huang

**Affiliations:** a College of Chemistry and Chemical Engineering, Xuchang University, Xuchang, Henan Province 461000, People’s Republic of China; b Institute of Surface Micro and Nano Materials, Xuchang University, Xuchang, Henan Province 461000, People’s Republic of China

## Abstract

In the title compound, C_16_H_12_F_4_N_2_O_3_S, the pyrimidine ring adopts a half-chair conformation; the mean plane formed by the ring atoms excluding the C atom bonded to the thio­phen-2-ylcarbonyl group has an r.m.s. deviation of 0.059 Å. The dihedral angle between the benzene and thio­phene rings is 62.26 (7)°. The mol­ecular conformation is stabilized by an intra­molecular O—H⋯O hydrogen bond, generating an *S*(6) ring. In the crystal, adjacent mol­ecules are connected *via* a centrosymmetric *R*_2_^2^(6) motif, formed by N—H⋯O hydrogen bonds.

## Related literature

For the bioactivity of dihydro­pyrimidines, see: Cochran *et al.* (2005 ▶); Zorkun *et al.* (2006 ▶); Moran *et al.* (2007 ▶). For the bioactivity of organofluorine compounds, see: Hermann *et al.* (2003 ▶); Ulrich (2004 ▶). For a related structure, see: Mosslemin *et al.* (2009 ▶).
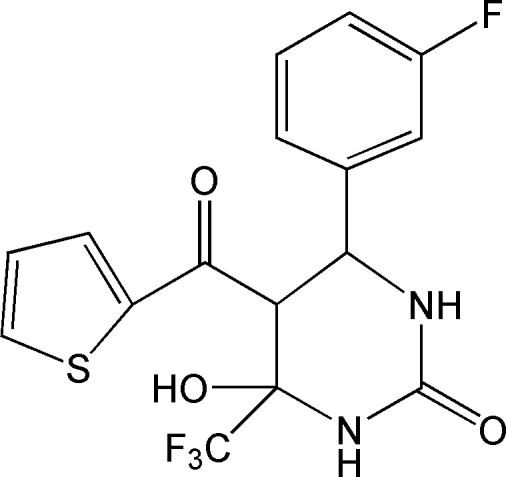


## Experimental

### 

#### Crystal data

C_16_H_12_F_4_N_2_O_3_S
*M* *_r_* = 388.34
Triclinic, 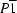

*a* = 6.6032 (10) Å
*b* = 10.4541 (16) Å
*c* = 12.4906 (18) Å
α = 77.136 (12)°
β = 78.940 (13)°
γ = 72.839 (11)°
*V* = 795.8 (2) Å^3^
*Z* = 2
Mo *K*α radiation
μ = 0.27 mm^−1^
*T* = 113 K
0.18 × 0.06 × 0.06 mm

#### Data collection

Rigaku Saturn CCD area-detector diffractometer
Absorption correction: multi-scan (*CrystalClear*; Rigaku, 2009 ▶) *T*_min_ = 0.953, *T*_max_ = 0.984
10393 measured reflections
3784 independent reflections
2531 reflections with *I* > 2σ(*I*)
*R*_int_ = 0.045

#### Refinement

*R*[*F* ^2^ > 2σ(*F* ^2^)] = 0.038
*wR*(*F* ^2^) = 0.082
*S* = 0.95
3784 reflections
247 parameters
H atoms treated by a mixture of independent and constrained refinement
Δρ_max_ = 0.36 e Å^−3^
Δρ_min_ = −0.24 e Å^−3^

### 

Data collection: *CrystalClear* (Rigaku, 2009 ▶); cell refinement: *CrystalClear*; data reduction: *CrystalClear*; program(s) used to solve structure: *SHELXS97* (Sheldrick, 2008 ▶); program(s) used to refine structure: *SHELXL97* (Sheldrick, 2008 ▶); molecular graphics: *SHELXTL* (Sheldrick, 2008 ▶); software used to prepare material for publication: *CrystalStructure* (Rigaku, 2009 ▶).

## Supplementary Material

Crystal structure: contains datablocks global, I. DOI: 10.1107/S1600536811006933/nk2083sup1.cif

Structure factors: contains datablocks I. DOI: 10.1107/S1600536811006933/nk2083Isup2.hkl

Additional supplementary materials:  crystallographic information; 3D view; checkCIF report

## Figures and Tables

**Table 1:** Hydrogen-bond geometry (Å, °)

*D*—H⋯*A*	*D*—H	H⋯*A*	*D*⋯*A*	*D*—H⋯*A*
O2—H2⋯O1	0.79 (2)	2.38 (2)	2.9609 (18)	131.7 (19)
N1—H1⋯O3^i^	0.858 (19)	1.99 (2)	2.851 (2)	175.9 (18)

Symmetry code: (i) 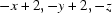.

**Fig. 1.**
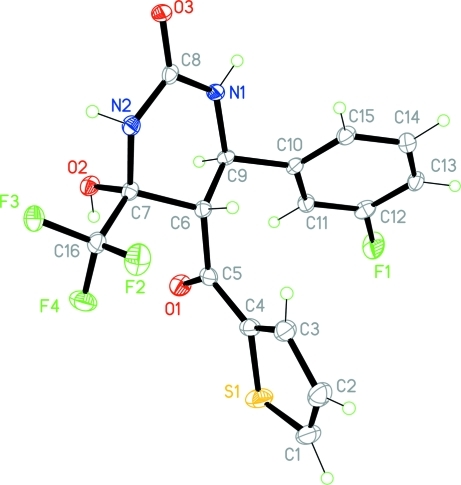
Molecular configuration and atom numbering scheme for (I), with displacement ellipsoids drawn at the 50% probability level.

## supplementary crystallographic information

### Comment

Dihydropyrimidine (DHPM) derivatives can be used as potential calcium channel blockers (Zorkun *et al.*, 2006), inhibitors of mitotic kinesin Eg5 for treating cancer (Cochran *et al.*, 2005) and as TRPA1 modulators for treating pain (Moran *et al.*, 2007). In addition, compounds that contain fluorine have special bioactivity, *e.g.* flumioxazin is a widely used herbicide (Hermann *et al.*, 2003; Ulrich, 2004). This led us to focus our attention on the synthesis and bioactivity of these important fused perfluoroalkylated heterocyclic compounds. During the synthesis of DHPM derivatives, the title compound, an intermediate C_16_H_12_F_4_N_2_O_3_S (I) was isolated and the structure confirmed by X-ray diffraction.

In the structure of the title compound, the dihydropyrimidine ring adopts a half-chair conformation with the C7/C8/C9/N1/N2 are nearly coplanar. The dihedral angle is 53.77 (5) ° between the dihydropyrimidine rings and the phenyl rings, and 80.57 (6) ° between the dihydropyrimidine rings and thiophene rings, respectively. The dihedral angle between the phenyl rings and thiophene rings is 62.26 (7) °. The molecular conformation is stabilized by intramolecular O—H···O hydrogen bond, generating an S(6) ring. In the crystal, adjacent molecules are connected via a centrosymmetric R^2^_2_(6) motif, formed by N—H···O hydrogen bonds. For a crystal structure related to the title compound, see: Mosslemin *et al.*, 2009.

### Experimental

The title compound was synthesized refluxing for 3 h a stirred solution of 3-fluorobenzaldehyde (0.24 g, 2 mmol), 4,4,4-trifluoro-1- (thiophen-2-yl)butane-1,3-dione (0.51 g, 2.3 mmol) and urea (0.18 g, 3 mmol) in 3 ml of anhydrous ethanol, the reaction catalyzed by sulfamic acid (0.06 g). The solvent was evaporated *in vacuo* and the residue was washed with water. The title compound was recrystallized from 50% aqueous ethanol and single crystals of (I) were obtained by slow evaporation.

### Refinement

Hydrogen atoms involved in hydrogen-bonding interactions were located by difference methods and their positional and isotropic displacement parameters were refined. Other H atoms were placed in calculated positions, with C–H(aromatic) = 0.95 Å and C–H(aliphatic) = 1.00 Å, and treated as riding, with *U*_iso_(H) = 1.2*U*eq(C).

### Crystal data

**Table:**

C_16_H_12_F_4_N_2_O_3_S	*Z* = 2
*M**_r_* = 388.34	*F*(000) = 396
Triclinic, *P*1	*D*_x_ = 1.621 Mg m^−^^3^
Hall symbol: -P 1	Mo *K*α radiation, λ = 0.71073 Å
*a* = 6.6032 (10) Å	Cell parameters from 3248 reflections
*b* = 10.4541 (16) Å	θ = 1.7–31.2°
*c* = 12.4906 (18) Å	µ = 0.27 mm^−^^1^
α = 77.136 (12)°	*T* = 113 K
β = 78.940 (13)°	Prism, colourless
γ = 72.839 (11)°	0.18 × 0.06 × 0.06 mm
*V* = 795.8 (2) Å^3^	

### Data collection

**Table:**

Rigaku Saturn CCD area-detector diffractometer	3784 independent reflections
Radiation source: rotating anode	2531 reflections with *I* > 2σ(*I*)
multilayer	*R*_int_ = 0.045
Detector resolution: 14.63 pixels mm^-1^	θ_max_ = 27.9°, θ_min_ = 1.7°
ω and φ scans	*h* = −8→8
Absorption correction: multi-scan (*CrystalClear*; Rigaku, 2009)	*k* = −13→13
*T*_min_ = 0.953, *T*_max_ = 0.984	*l* = −16→16
10393 measured reflections	

### Refinement

**Table:**

Refinement on *F*^2^	Primary atom site location: structure-invariant direct methods
Least-squares matrix: full	Secondary atom site location: difference Fourier map
*R*[*F*^2^ > 2σ(*F*^2^)] = 0.038	Hydrogen site location: inferred from neighbouring sites
*wR*(*F*^2^) = 0.082	H atoms treated by a mixture of independent and constrained refinement
*S* = 0.95	*w* = 1/[σ^2^(*F*_o_^2^) + (0.0352*P*)^2^] where *P* = (*F*_o_^2^ + 2*F*_c_^2^)/3
3784 reflections	(Δ/σ)_max_ = 0.001
247 parameters	Δρ_max_ = 0.36 e Å^−^^3^
0 restraints	Δρ_min_ = −0.24 e Å^−^^3^

### Special details

**Table:**

Geometry. All e.s.d.'s (except the e.s.d. in the dihedral angle between two l.s. planes) are estimated using the full covariance matrix. The cell e.s.d.'s are taken into account individually in the estimation of e.s.d.'s in distances, angles and torsion angles; correlations between e.s.d.'s in cell parameters are only used when they are defined by crystal symmetry. An approximate (isotropic) treatment of cell e.s.d.'s is used for estimating e.s.d.'s involving l.s. planes.
Refinement. Refinement of *F*^2^ against ALL reflections. The weighted *R*-factor *wR* and goodness of fit *S* are based on *F*^2^, conventional *R*-factors *R* are based on *F*, with *F* set to zero for negative *F*^2^. The threshold expression of *F*^2^ > σ(*F*^2^) is used only for calculating *R*-factors(gt) *etc*. and is not relevant to the choice of reflections for refinement. *R*-factors based on *F*^2^ are statistically about twice as large as those based on *F*, and *R*- factors based on ALL data will be even larger.

### Fractional atomic coordinates and isotropic or equivalent isotropic displacement parameters (Å^2^)

**Table:**

	*x*	*y*	*z*	*U*_iso_*/*U*_eq_	
S1	0.10178 (8)	0.67205 (5)	0.43354 (4)	0.02686 (14)	
F1	0.54415 (17)	0.42910 (11)	0.11049 (10)	0.0313 (3)	
F2	0.38941 (17)	1.06273 (11)	0.41921 (8)	0.0275 (3)	
F3	0.26149 (16)	1.25835 (10)	0.32224 (9)	0.0227 (3)	
F4	0.09036 (16)	1.10363 (11)	0.35651 (9)	0.0267 (3)	
O1	0.15491 (18)	0.87185 (12)	0.22648 (10)	0.0199 (3)	
O2	0.2727 (2)	1.13183 (13)	0.14128 (10)	0.0167 (3)	
H2	0.178 (3)	1.097 (2)	0.1497 (18)	0.036 (7)*	
O3	0.89825 (18)	1.13035 (12)	0.07872 (10)	0.0176 (3)	
N1	0.7582 (2)	0.95351 (15)	0.08904 (13)	0.0146 (3)	
N2	0.5923 (2)	1.12219 (16)	0.19491 (12)	0.0153 (3)	
C1	0.1989 (3)	0.5998 (2)	0.55599 (16)	0.0278 (5)	
H1A	0.1369	0.5394	0.6120	0.033*	
C2	0.3737 (3)	0.63720 (19)	0.56549 (16)	0.0254 (5)	
H2B	0.4474	0.6065	0.6286	0.031*	
C3	0.4332 (3)	0.72758 (18)	0.46990 (15)	0.0198 (4)	
H3	0.5523	0.7640	0.4615	0.024*	
C4	0.3001 (3)	0.75680 (18)	0.39064 (15)	0.0174 (4)	
C5	0.2979 (3)	0.84873 (17)	0.28367 (15)	0.0152 (4)	
C6	0.4813 (3)	0.91633 (17)	0.24203 (14)	0.0131 (4)	
H6	0.5862	0.8833	0.2966	0.016*	
C7	0.4045 (3)	1.07322 (17)	0.22502 (14)	0.0130 (4)	
C8	0.7569 (3)	1.07005 (17)	0.11741 (14)	0.0140 (4)	
C9	0.5952 (3)	0.87853 (17)	0.12902 (14)	0.0130 (4)	
H9	0.4880	0.9089	0.0757	0.016*	
C10	0.6952 (3)	0.72647 (17)	0.13508 (14)	0.0135 (4)	
C11	0.5744 (3)	0.64573 (18)	0.11689 (15)	0.0161 (4)	
H11	0.4320	0.6846	0.1005	0.019*	
C12	0.6671 (3)	0.50868 (18)	0.12332 (15)	0.0187 (4)	
C13	0.8746 (3)	0.44660 (18)	0.14207 (15)	0.0196 (4)	
H13	0.9345	0.3518	0.1434	0.024*	
C14	0.9932 (3)	0.52851 (18)	0.15906 (15)	0.0203 (4)	
H14	1.1375	0.4893	0.1722	0.024*	
C15	0.9039 (3)	0.66661 (18)	0.15703 (14)	0.0168 (4)	
H15	0.9860	0.7207	0.1708	0.020*	
C16	0.2862 (3)	1.12363 (18)	0.33183 (15)	0.0174 (4)	
H2A	0.572 (3)	1.207 (2)	0.1936 (16)	0.021 (5)*	
H1	0.862 (3)	0.9241 (19)	0.0400 (16)	0.023 (6)*	

### Atomic displacement parameters (Å^2^)

**Table:**

	*U*^11^	*U*^22^	*U*^33^	*U*^12^	*U*^13^	*U*^23^
S1	0.0281 (3)	0.0272 (3)	0.0271 (3)	−0.0167 (2)	0.0034 (2)	−0.0019 (2)
F1	0.0319 (7)	0.0164 (6)	0.0517 (8)	−0.0106 (5)	−0.0110 (6)	−0.0081 (5)
F2	0.0390 (7)	0.0251 (6)	0.0174 (6)	−0.0029 (5)	−0.0083 (5)	−0.0050 (5)
F3	0.0261 (6)	0.0143 (5)	0.0276 (6)	−0.0042 (5)	0.0008 (5)	−0.0086 (5)
F4	0.0218 (6)	0.0281 (6)	0.0318 (6)	−0.0120 (5)	0.0092 (5)	−0.0122 (5)
O1	0.0183 (7)	0.0207 (7)	0.0236 (7)	−0.0083 (6)	−0.0065 (6)	−0.0020 (6)
O2	0.0158 (7)	0.0149 (7)	0.0209 (7)	−0.0060 (6)	−0.0054 (6)	−0.0011 (5)
O3	0.0141 (6)	0.0139 (6)	0.0258 (7)	−0.0070 (5)	0.0009 (5)	−0.0041 (6)
N1	0.0133 (8)	0.0118 (8)	0.0193 (8)	−0.0052 (6)	0.0008 (7)	−0.0041 (6)
N2	0.0151 (8)	0.0112 (8)	0.0208 (8)	−0.0045 (7)	−0.0011 (6)	−0.0052 (7)
C1	0.0352 (12)	0.0204 (10)	0.0217 (10)	−0.0081 (9)	0.0087 (9)	−0.0009 (8)
C2	0.0341 (12)	0.0215 (10)	0.0170 (10)	−0.0047 (9)	−0.0016 (9)	−0.0008 (8)
C3	0.0221 (10)	0.0180 (10)	0.0191 (10)	−0.0067 (8)	−0.0010 (8)	−0.0023 (8)
C4	0.0178 (9)	0.0158 (9)	0.0189 (9)	−0.0069 (8)	0.0029 (8)	−0.0052 (8)
C5	0.0156 (9)	0.0122 (9)	0.0187 (9)	−0.0042 (8)	0.0017 (8)	−0.0070 (7)
C6	0.0123 (9)	0.0115 (9)	0.0161 (9)	−0.0039 (7)	−0.0028 (7)	−0.0020 (7)
C7	0.0143 (9)	0.0114 (8)	0.0149 (9)	−0.0049 (7)	−0.0039 (7)	−0.0021 (7)
C8	0.0136 (9)	0.0115 (9)	0.0167 (9)	−0.0028 (7)	−0.0062 (7)	0.0005 (7)
C9	0.0127 (9)	0.0118 (9)	0.0159 (9)	−0.0066 (7)	−0.0012 (7)	−0.0017 (7)
C10	0.0172 (9)	0.0114 (9)	0.0118 (8)	−0.0048 (8)	−0.0007 (7)	−0.0014 (7)
C11	0.0147 (9)	0.0137 (9)	0.0205 (9)	−0.0042 (7)	−0.0034 (8)	−0.0029 (7)
C12	0.0243 (10)	0.0157 (9)	0.0211 (10)	−0.0124 (8)	−0.0032 (8)	−0.0036 (8)
C13	0.0233 (10)	0.0116 (9)	0.0198 (10)	−0.0019 (8)	0.0011 (8)	−0.0010 (8)
C14	0.0162 (10)	0.0177 (10)	0.0242 (10)	−0.0024 (8)	−0.0034 (8)	0.0001 (8)
C15	0.0179 (10)	0.0153 (9)	0.0182 (9)	−0.0068 (8)	−0.0039 (8)	−0.0002 (8)
C16	0.0177 (10)	0.0124 (9)	0.0220 (10)	−0.0041 (8)	−0.0026 (8)	−0.0028 (8)

### Geometric parameters (Å, °)

**Table:**

S1—C1	1.702 (2)	C3—C4	1.374 (2)
S1—C4	1.7293 (18)	C3—H3	0.9500
F1—C12	1.3730 (19)	C4—C5	1.462 (2)
F2—C16	1.335 (2)	C5—C6	1.528 (2)
F3—C16	1.3495 (19)	C6—C9	1.543 (2)
F4—C16	1.3372 (19)	C6—C7	1.545 (2)
O1—C5	1.228 (2)	C6—H6	1.0000
O2—C7	1.406 (2)	C7—C16	1.529 (2)
O2—H2	0.79 (2)	C9—C10	1.521 (2)
O3—C8	1.2414 (19)	C9—H9	1.0000
N1—C8	1.340 (2)	C10—C15	1.388 (2)
N1—C9	1.462 (2)	C10—C11	1.397 (2)
N1—H1	0.858 (19)	C11—C12	1.373 (2)
N2—C8	1.377 (2)	C11—H11	0.9500
N2—C7	1.433 (2)	C12—C13	1.372 (2)
N2—H2A	0.855 (19)	C13—C14	1.390 (2)
C1—C2	1.356 (3)	C13—H13	0.9500
C1—H1A	0.9500	C14—C15	1.386 (2)
C2—C3	1.417 (2)	C14—H14	0.9500
C2—H2B	0.9500	C15—H15	0.9500
			
C1—S1—C4	91.22 (10)	C16—C7—C6	111.98 (14)
C7—O2—H2	111.1 (16)	O3—C8—N1	123.00 (17)
C8—N1—C9	126.08 (16)	O3—C8—N2	119.39 (16)
C8—N1—H1	115.4 (13)	N1—C8—N2	117.60 (16)
C9—N1—H1	118.4 (13)	N1—C9—C10	110.62 (14)
C8—N2—C7	121.26 (15)	N1—C9—C6	107.74 (14)
C8—N2—H2A	113.8 (13)	C10—C9—C6	112.89 (14)
C7—N2—H2A	115.5 (12)	N1—C9—H9	108.5
C2—C1—S1	113.23 (15)	C10—C9—H9	108.5
C2—C1—H1A	123.4	C6—C9—H9	108.5
S1—C1—H1A	123.4	C15—C10—C11	119.36 (16)
C1—C2—C3	111.78 (18)	C15—C10—C9	121.52 (15)
C1—C2—H2B	124.1	C11—C10—C9	119.11 (15)
C3—C2—H2B	124.1	C12—C11—C10	118.21 (16)
C4—C3—C2	112.76 (17)	C12—C11—H11	120.9
C4—C3—H3	123.6	C10—C11—H11	120.9
C2—C3—H3	123.6	C13—C12—C11	123.99 (17)
C3—C4—C5	130.49 (16)	C13—C12—F1	118.16 (16)
C3—C4—S1	111.01 (14)	C11—C12—F1	117.84 (16)
C5—C4—S1	118.45 (14)	C12—C13—C14	117.07 (17)
O1—C5—C4	121.99 (16)	C12—C13—H13	121.5
O1—C5—C6	119.49 (16)	C14—C13—H13	121.5
C4—C5—C6	118.52 (15)	C15—C14—C13	120.89 (17)
C5—C6—C9	109.89 (14)	C15—C14—H14	119.6
C5—C6—C7	112.76 (14)	C13—C14—H14	119.6
C9—C6—C7	106.99 (13)	C14—C15—C10	120.42 (17)
C5—C6—H6	109.0	C14—C15—H15	119.8
C9—C6—H6	109.0	C10—C15—H15	119.8
C7—C6—H6	109.0	F2—C16—F4	107.48 (14)
O2—C7—N2	108.42 (14)	F2—C16—F3	106.62 (15)
O2—C7—C16	108.32 (14)	F4—C16—F3	106.94 (14)
N2—C7—C16	107.11 (14)	F2—C16—C7	112.78 (14)
O2—C7—C6	113.95 (14)	F4—C16—C7	111.68 (15)
N2—C7—C6	106.77 (14)	F3—C16—C7	111.02 (14)
			
C4—S1—C1—C2	0.06 (16)	C8—N1—C9—C6	−24.5 (2)
S1—C1—C2—C3	−0.2 (2)	C5—C6—C9—N1	175.83 (13)
C1—C2—C3—C4	0.4 (2)	C7—C6—C9—N1	53.10 (17)
C2—C3—C4—C5	176.81 (18)	C5—C6—C9—C10	−61.73 (18)
C2—C3—C4—S1	−0.3 (2)	C7—C6—C9—C10	175.53 (13)
C1—S1—C4—C3	0.16 (15)	N1—C9—C10—C15	30.8 (2)
C1—S1—C4—C5	−177.37 (15)	C6—C9—C10—C15	−90.03 (19)
C3—C4—C5—O1	−173.57 (18)	N1—C9—C10—C11	−148.51 (15)
S1—C4—C5—O1	3.4 (2)	C6—C9—C10—C11	90.68 (19)
C3—C4—C5—C6	7.1 (3)	C15—C10—C11—C12	1.0 (3)
S1—C4—C5—C6	−175.94 (13)	C9—C10—C11—C12	−179.65 (16)
O1—C5—C6—C9	−57.9 (2)	C10—C11—C12—C13	−2.8 (3)
C4—C5—C6—C9	121.40 (17)	C10—C11—C12—F1	177.01 (15)
O1—C5—C6—C7	61.3 (2)	C11—C12—C13—C14	2.2 (3)
C4—C5—C6—C7	−119.34 (17)	F1—C12—C13—C14	−177.57 (16)
C8—N2—C7—O2	−78.04 (19)	C12—C13—C14—C15	0.0 (3)
C8—N2—C7—C16	165.27 (15)	C13—C14—C15—C10	−1.7 (3)
C8—N2—C7—C6	45.1 (2)	C11—C10—C15—C14	1.1 (3)
C5—C6—C7—O2	−64.89 (19)	C9—C10—C15—C14	−178.19 (16)
C9—C6—C7—O2	56.04 (18)	O2—C7—C16—F2	173.58 (13)
C5—C6—C7—N2	175.45 (14)	N2—C7—C16—F2	−69.67 (18)
C9—C6—C7—N2	−63.62 (17)	C6—C7—C16—F2	47.07 (19)
C5—C6—C7—C16	58.50 (19)	O2—C7—C16—F4	52.41 (18)
C9—C6—C7—C16	179.43 (14)	N2—C7—C16—F4	169.17 (13)
C9—N1—C8—O3	−178.49 (15)	C6—C7—C16—F4	−74.09 (18)
C9—N1—C8—N2	2.9 (2)	O2—C7—C16—F3	−66.84 (18)
C7—N2—C8—O3	167.11 (15)	N2—C7—C16—F3	49.92 (18)
C7—N2—C8—N1	−14.3 (2)	C6—C7—C16—F3	166.66 (14)
C8—N1—C9—C10	−148.29 (16)		

### Hydrogen-bond geometry (Å, °)

**Table:**

*D*—H···*A*	*D*—H	H···*A*	*D*···*A*	*D*—H···*A*
O2—H2···O1	0.79 (2)	2.38 (2)	2.9609 (18)	131.7 (19)
N1—H1···O3^i^	0.858 (19)	1.99 (2)	2.851 (2)	175.9 (18)

Symmetry codes: (i) −*x*+2, −*y*+2, −*z*.

## References

[bb1] Cochran, J. C., Gatial, J. E., Kapoor, T. M. & Gilbert, S. P. (2005). *J. Biol. Chem.* **280**, 12658–12667. 10.1074/jbc.M413140200 PMC1356610 15665380

[bb2] Hermann, B., Erwin, H. & Hansjorg, K. (2003). US Patent No. 2 003 176 284.

[bb3] Moran, M. M., Fanger, C., Chong, J. A., McNamara, C., Zhen, X. G. & Mandel-Brehm, J. (2007). WO Patent No. 2 007 073 505.

[bb4] Mosslemin, M. H., Nateghi, M. R., Sadoughi, H. & Lamei, A. (2009). *Acta Cryst.* E**65**, o1339. 10.1107/S1600536809017097 PMC2969779 21583192

[bb5] Rigaku (2009). *CrystalClear* and *CrystalStructure* Rigaku/MSC, The Woodlands, Texas, USA.

[bb6] Sheldrick, G. M. (2008). *Acta Cryst.* A**64**, 112–122. 10.1107/S0108767307043930 18156677

[bb7] Ulrich, H. (2004). US Patent No. 2 004 033 897.

[bb8] Zorkun, I. S., Sarac, S., Celebi, S. & Erol, K. (2006). *Bioorg. Med. Chem.* **14**, 8582–8589. 10.1016/j.bmc.2006.08.031 16971126

